# Human ex vivo lung perfusion: a novel model to study human lung diseases

**DOI:** 10.1038/s41598-020-79434-4

**Published:** 2021-01-12

**Authors:** Nayra Cárdenes, John Sembrat, Kentaro Noda, Tyler Lovelace, Diana Álvarez, Humberto E. Trejo Bittar, Brian J. Philips, Mehdi Nouraie, Panayiotis V. Benos, Pablo G. Sánchez, Mauricio Rojas

**Affiliations:** 1 Dorothy P. and Richard P. Simmons Center for Interstitial Lung Disease, University of Pittsburgh School of Medicine, W1244 BST Tower, 200 Lothrop Street, Pittsburgh, PA 15261 USA; 2 Division of Pulmonary, Allergy and Critical Care Medicine, University of Pittsburgh School of Medicine, Pittsburgh, PA USA; 3 Division of Lung Transplant and Lung Failure, Department of Cardiothoracic Surgery, University of Pittsburgh Medical Center, Pittsburgh, PA USA; 4 Department of Computational Biology, University of Pittsburgh, Pittsburgh, PA USA; 5 Joint CMU-Pitt Ph.D. Program in Computational Biology, Pittsburgh, PA USA; 6 Department of Pathology, Thoracic and Autopsy Pathology, University of Pittsburgh Medical Center, Pittsburgh, PA USA; 7 Clinical and Translational Science Institute, University of Pittsburgh, Pittsburgh, PA USA

**Keywords:** Medical research, Preclinical research

## Abstract

Experimental animal models to predict physiological responses to injury and stress in humans have inherent limitations. Therefore, the development of preclinical human models is of paramount importance. Ex vivo lung perfusion (EVLP) has typically been used to recondition donor lungs before transplantation. However, this technique has recently advanced into a model to emulate lung mechanics and physiology during injury. In the present study, we propose that the EVLP of diseased human lungs is a well-suited preclinical model for translational research on chronic lung diseases. Throughout this paper, we demonstrate this technique's feasibility in pulmonary arterial hypertension (PAH), idiopathic pulmonary fibrosis (IPF), emphysema, and non-disease donor lungs not suitable for transplantation. In this study, we aimed to perfuse the lungs for 6 h with the EVLP system. This facilitated a robust and continuous assessment of airway mechanics, pulmonary hemodynamics, gas exchange, and biochemical parameters. We then collected at different time points tissue biopsies of lung parenchyma to isolate RNA and DNA to identify each disease's unique gene expression. Thus, demonstrating that EVLP could successfully serve as a clinically relevant experimental model to derive essential insights into pulmonary pathophysiology and various human lung diseases.

## Introduction

Chronic lung diseases are the third leading cause of disease-related death in the US^1^. Murine models are among the few available tools in pulmonary diseases used as a platform for preclinical trials^2–8^. Despite similar genomic compositions, innate and adaptive immune responses in disease conditions vary widely between humans and mice^9,10^. Additionally, the utilization of such models has severe limitations. The use of bleomycin to induce pulmonary fibrosis is well recognized as a flawed model to reflect the changes observed in idiopathic pulmonary fibrosis (IPF). Similar limitations in murine models have been noted for emphysema and pulmonary arterial hypertension (PAH). For example, Seok et al*.* reported that human septic patients with significant proinflammatory responses showed marked differences in gene expression patterns compared to well-established small animal models of inflammation^10^. To address this unmet need in the field of translational research, we, along with others, have used EVLP as a preclinical model by having endotoxin-induced acute lung injury to evaluate novel pharmacologic and cell therapy interventions^11–16^.

Throughout this study, we successfully observed the novel systematic integration of lung mechanics and the characterization of the cellular and molecular changes during ex vivo lung perfusion (EVLP) of chronic lung diseases. The physiological, biochemical, histopathological, and genomic data acquired from this unique experimental system could eventually be utilized as a platform to perform translational research in specific and more realistic disease models.

## Methods

### Ex-vivo lung perfusion (EVLP)

**Figure 1:**
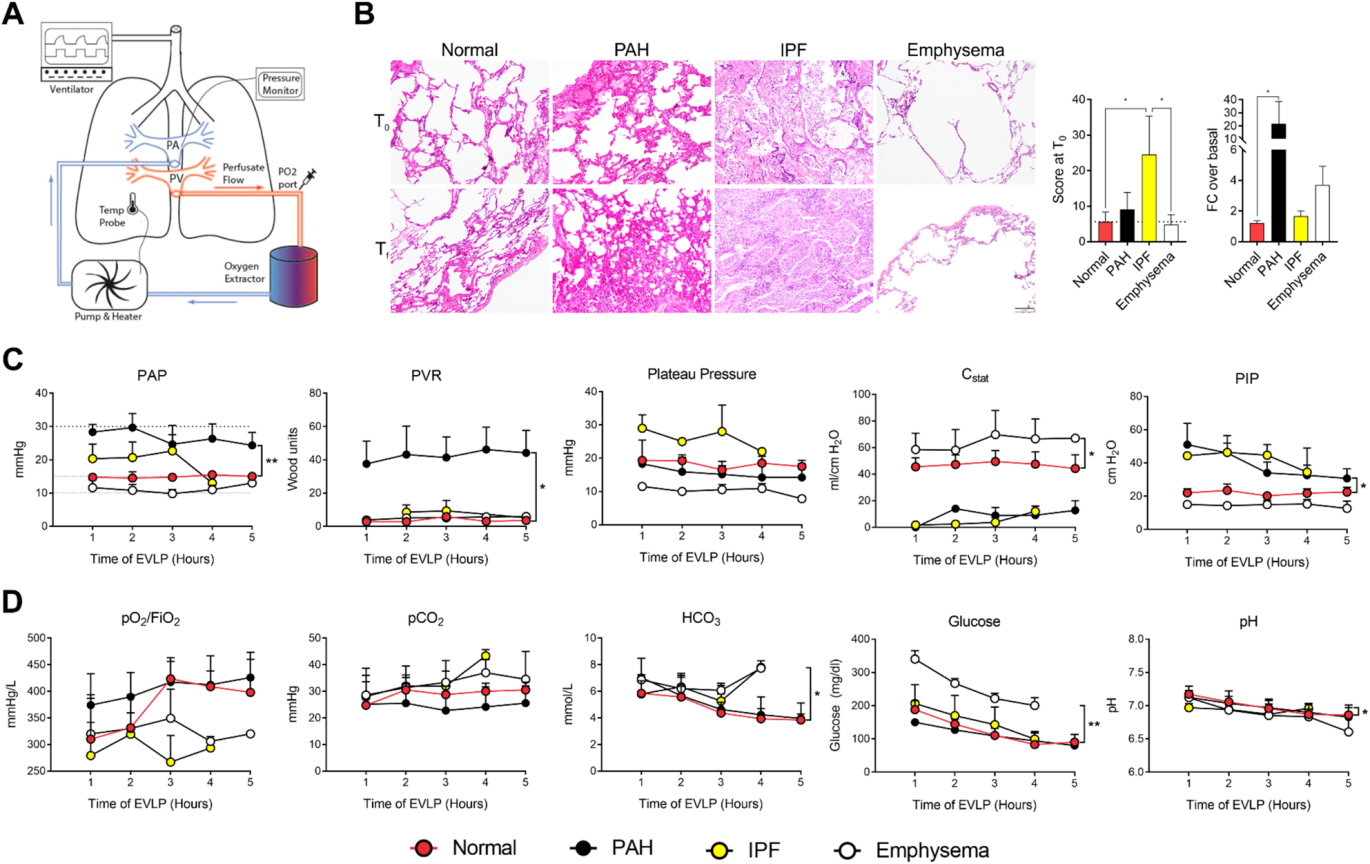
Human ex vivo lung perfusion. (**A**) Schematic of the experimental setup (previously published)^11^. (**B**) Kinetic analysis of Histopathology from Normal, PAH, Emphysema, and IPF Lungs during Ex Vivo Lung Perfusion (EVLP). Representative hematoxylin and eosin staining (20x) high-power magnification of lung parenchyma biopsy specimens at the beginning (0 h) and end of perfusion (6 h) shows maintenance of lung architecture. Quantification of the blind scoring of histology images is shown in bar graphs for time 0 and as fold change (FC) of end of EVLP over start. (**C**) Pulmonary artery pressure, PVR, peak Airway pressure, static lung compliance, and Peak Inspiratory Pressure throughout EVLP. (**D**) Gas exchange parameters including pO_2_, pCO_2,_ HCO_3_, and pH and biochemical assessment of lung metabolism by measurement of glucose throughout perfusion in normal (red), emphysema (white), IPF (grey), and PAH (black) lungs at various timepoints on EVLP. *p < 0.05; **p < 0.01.

All methods utilized during this study were approved according to guidelines defined by the Committee for Oversight of Research and Clinical Training Involving Decedents (CORID) (protocols #101 and 401) at the University of Pittsburgh. The EVLP technique has been previously defined^11,16–18^, and the Institutional Biosafety Committee approved our experimental protocols before any experiment. Following informed consent signed by an authorized next of kin, lungs not suitable for clinical use were procured under an established and approved CORID protocol. Lungs from IPF, emphysema, PAH, and healthy donors were procured as per the standard protocol^19^ and then preserved using cold static preservation technique^20,21^. The perfusion system primed with 1.5 L of STEEN Solution™ (XVIVO Perfusion AB) was infused with Heparin, Cefazolin, and Solu-Medrol. The XVIVO cannula set pulmonary artery and pulmonary vein were placed surgically and then connected on the EVLP system, as illustrated in Fig. 1A. The standardized EVLP protocol for healthy lungs has been described previously^22–24^. During the study, we ventilated PHA lungs with a positive end-expiratory pressure (PEEP) 5 cm H_2_O, respiratory rate (RR) 7 bpm, tidal volume (TV) 7 ml/kg, inspiratory: expiratory ratio (I:E) 1:2, and FiO_2_ 21%. For emphysema lungs, ventilator settings were tidal volume (TV): 6 ml/kg, positive end-expiratory pressure (PEEP): 3 cmH_**2**_O, respiratory rate (RR): 8 bpm, and inspiratory: expiratory ratio (I:E): 1:4 emphysema, and for IPF lungs TV: 4–6 ml/kg, PEEP: 12–15 cmH_**2**_O, RR: 15 bpm and I:E: 3:1 for IPF. For the hourly lung evaluations, lungs were ventilated with 100% O_2_ with a TV 10 ml/kg for 10 min. Subsequently, we measured physiologic vascular and respiratory parameters and collected perfusate for biochemical analysis and assessment of gas exchange (pO_2_, pCO_2_, and pH). We then collected tissue biopsies at 0, 3, and 6 h for RNA and histologic analysis.

### Measurement of cytokines in perfusion solution

The concentrations of cytokines in the perfusion solution were measured using multiplexed bead-based immunoassays (Millipore, USA) at the UPMC Cancer Biomarkers Facility: Luminex Core Laboratory. Hourly perfusate samples were collected, centrifuged at 10,000 RPM, and supernatant stored at -80 °C until further processing. Bio-Data was analyzed in duplicate using the Bio-Rad BioPlex Manager Software version 4.1.1, and concentrations were expressed in pg/ml.

### Quantitation of mitochondrial DNA (mtDNA) in perfusion solution

200 μl of perfusate samples were processed to isolate DNA using the Qiagen DNeasy Blood and Tissue kit (Qiagen, USA). qRT-PCR was performed to measure mtDNA utilizing SYBR Green PCR mix (Applied Biosystems, USA) and primers for mitochondria genes; human NADH dehydrogenase (hND) and human COXIII. mtDNA contents were normalized with 18SrRNA nuclear DNA.

### Histology

A 1.5 by 1.0-in. lung biopsy, mainly sourced from the upper lobe of both lungs, was collected at different time points and divided into small sections to complete histological, RNA, and protein expression. For histology, specimens were embedded in paraffin after fixation in 10% buffered formalin for 24 h, followed by 5 mm thick sectioning and hematoxylin and eosin staining**.** Blinded review for lung injury (pre and post-EVLP) was done by a lung pathologist, using a histological scoring system considering interstitial or alveolar neutrophil infiltration, hyaline membrane or proteinaceous debris filling the airspaces, and alveolar septal thickening following the recommendation by Matute-Bello et al*.*^25^ and taking into account the contribution of heterogeneity of the sample (Table 2).

### RNA sequencing

A section of 0.5 square inches from the total tissue biopsy collected from disease and healthy lungs, as previously described, was immediately flash-frozen in liquid nitrogen, unto RNA was isolated via the Trizol method (Invitrogen, USA) as previously described^11^. Concentrations were determined using Qubit 2.0 fluorometer and Agilent TapeStation 2200 for RNA quantity and quality (Agilent, USA). Samples were sequenced on an Illumina NextSeq 500′s platform by the Health Sciences Sequencing Core at Children’s Hospital of Pittsburgh. Raw transcript data was processed by Salmon^26^, which mapped reads to the reference genome (GRCh38) and calculated the transcripts per million (TPMs). Differentially expressed genes (DEGs) were determined using Sleuth^27^ at a false discovery rate (FDR) ≤ 0.05. Pathway significance assessment was done using Fisher’s exact test through the Gene Set Enrichment Analysis (GSEA)^28,29^. This publication's data have been deposited in NCBI's Gene Expression Omnibus (GEO) and are accessible via GEO series number GSE146436.

### Statistical analysis

All *p* values were calculated by a mixed-effect model with a random coefficient effect and Restricted Maximum Likelihood method. The p value indicates different time trends of response between groups using time and group interaction in each model. If the p value for interaction was ≥ 0.2, the interaction term was removed from the model. All p values were calculated using the bootstrapping method, and all analyses performed in STATA 14.0 (StataCorp., College Station, TX).

## Results

### Demographics

In the present study, a total of 5 non-disease human lungs from donors non-suitable for transplantation and 11 disease lungs were evaluated longitudinally for 4–6 h on EVLP for physiological, biochemical, histological, and genetic analysis. For comparison, lungs from 3 PAH (females, mean age 46 yr ± 8 SD; mean BMI 39.4 kg/m^2^ ± 9.9 SD), five emphysema (3 males and two females, mean age 56 yr ± 10 SD; mean BMI 24.0 kg/m^2^ ± 7.6 SD), 3 IPF donors (2 males and one female, mean age 68 yr ± 11 SD; mean BMI 35.9 kg/m^2^ ± 4.3 SD) and five healthy donors (2 females and three males; mean age 59 yr ± 11 SD; mean BMI 33.9 kg/m^2^ ± 8.3 SD) were included in this study (Table 1). The donor demographics data obtained from the organ procurement agency were de-identified and categorized, each representing the respective disease group without significant difference in age and BMI**.**

**Table 1:** Donor demographic data.

	Sex	Age	Height (m)	Weight (kg)	BMI
Normal	F	48	1.60	84	33
	F	58	1.63	119	45
	M	64	1.7	114	39
	M	51	1.78	88	28
	M	76	1.85	84	24
PAH	F	38	1.57	76	31
	F	46	1.67	104	37
	F	53	1.5	113	50
IPF	M	74	1.65	88	32
	F	55	1.68	111	39
	M	75	1.83	104	31
Emphysema	F	49	1.63	54	20
	F	46	1.57	47	19
	M	73	1.78	64	20
	M	55	1.65	102	37
	M	56	1.78	73	23

### Maintenance of parenchymal architecture on histopathology at the end of perfusion for healthy and diseased lung preparations

Histologic evaluation of lung biopsy specimens was performed and compared at the beginning and the end of EVLP. For this purpose, we utilized a lung histology scoring system (Table 2) described before^25^. Briefly, the inflammatory response from neutrophil infiltration within the alveolar space and interstitium, alveolar septal thickening, or damage leads to proteinaceous exudate and alveolar space edema was assessed in terms of heterogeneity of distribution (with one being homogeneous and four highly heterogeneous).

**Table 2:** Histopathological human lung injury scoring system.

Parameter	0	1	2
Neutrophils in the alveolar space	None	1–5	> 5
Neutrophils in the interstitial space	None	1–5	> 5
Hyaline Membranes	None	1	> 1
Proteinaceous debris filling the airspaces	None	1	> 1
Alveolar septal thickening	< 2x	2x–4x	> 4x

As expected, lungs presented with different basal states associated with their disease. We observed an increase in the disease states' proteinaceous debris, which was significantly higher in the PAH lungs (p = 0.007, compared to normal) and a significant alveolar septal thickening in the IPF lungs associated with the fibrosis (p = 0.007, compared to normal controls). Throughout EVLP, there was no significant overall change in the pre-/post- EVLP lung injury score in the healthy lungs, whereas the diseased lungs deteriorated over time, especially the PAH and the emphysema lungs (Fig. 1B). Whether this inflammatory response is due to EVLP per se or a result of lung recruitment needs to be determined.

### Differential changes in lung physiology for various disease groups on EVLP

Real-time and continuous measurement of airway mechanics and pulmonary hemodynamics on EVLP facilitated modulation of ventilatory and perfusion parameters catering to specific lung conditions. Multiple mechanical parameters were collected at multiple time points during a period of 4 to 6 h (Fig. 1C). At the onset of perfusion, mean pulmonary artery pressures (mPAP) were significantly elevated in PAH, whereas in IPF lungs, they were moderately elevated and within normal limits in emphysematous lungs. In severe PAH cases, mPAP can very well approach systemic pressures leading to poor right ventricular function and right heart failure culminating in dismal outcomes. Clinically, this is manifest as the development of pulmonary edema. Thereby, the elevated intrinsic PA pressures in the lungs with a particular PAH component dictated that flow rates be reduced to normalize the PA pressures. Our goal was to maintain PA pressures between 10–15 mmHg and left atrial pressures (LAP) in the range of 3 to 5 mm Hg to avoid hydrostatic damage to the lung during EVLP. LAP was controlled by adjusting the height of the reservoir. Pulmonary vascular resistance (PVR) is significantly elevated in patients with PAH and is pathognomonic of this condition^30^. As such, the measured PVR was higher in PAH lungs than in healthy lungs (42.63 dynes/sec/cm^5^ ± 2.93 SD *vs.* 10.49 dynes/sec/cm^5^ ± 4.1 SD). While rising PVR is an ominous sign and on the ex vivo system represents endothelial damage, in our experiment, both IPF and emphysema had low PVR, which was sustained for the entire period of perfusion.

The average peak inspiratory pressures (PIP) during assist controlled ventilation with a tidal volume of 6 ml/kg are usually < 30 cmH_2_O^30^ and depend on patient factors like negative inspiratory pressure and chest wall rigidity. For obvious reasons, these factors could not be simulated in this ex vivo lung preparation. PIP was profoundly elevated in IPF and PAH lungs at the beginning of EVLP, whereas they remained within normal range throughout the perfusion in emphysema and healthy lungs**.** Dynamic lung compliance (C_dyn_) was maintained in the normal range (50–100 ml/cmH_2_O) throughout the perfusion in emphysema and healthy lungs and was significantly lower in IPF and PAH lungs (data not shown). This correlates with observations seen in routine clinical practice. Corresponding findings were also noticed with static lung compliance (C_stat_) (Fig. 1C).

### Gas exchange and biochemical parameters for various lung diseases on EVLP

Hourly gas exchange was evaluated by measuring the partial pressure of oxygen (PO_2_), the partial pressure of carbon dioxide (PCO_2_), pH, glucose, and bicarbonate (HCO_3_) levels in the perfusate solution in the pulmonary arterial (PA) and venous (PV) ends (Fig. 1D). We used the perfusate returning from the lung (PV) for comparison within groups since it would represent the lung microenvironment**.** PaO_2_/FiO_2_ ratio was lower in the IPF and emphysema groups than PAH and healthy lungs and did not improve throughout perfusion. These values were within normal clinical range (PO_2_ average 80–90 mmHg with ambient O_2_; PO_2_/FiO_2_ average ratio > 200; PCO_2_ normal 34–47 mmHg). PCO_2_ was highest in emphysema and IPF lungs compared to PAH and healthy lungs. The persistently elevated CO_2_ in these groups warranted notable adjustment in ventilatory parameters. The respiratory rate was adjusted to 8 breaths per minute (bpm) and I:E of 1:4 for emphysema donor lungs, whereas for the IPF group, the rate had to be increased to 15 bpm, and I:E was set at 3:1. These adjustments were made to maintain lung physiology close to normal limits. We observed that the PAH lungs exhibit statistically significant reduced glucose consumption *p* = 0.012, and IPF was the only group that did not drop pH throughout the experiment. These changes probably could have been either due to adjusted flow through the ex vivo circuit to normalize PA pressures or variable metabolic rates in these lungs with limited glucose utilization. However, further investigation of these changes is needed. These findings may be correlated to cell metabolic changes during chronic disease.

### Determination of soluble markers of cellular damage.

**Figure 2:**
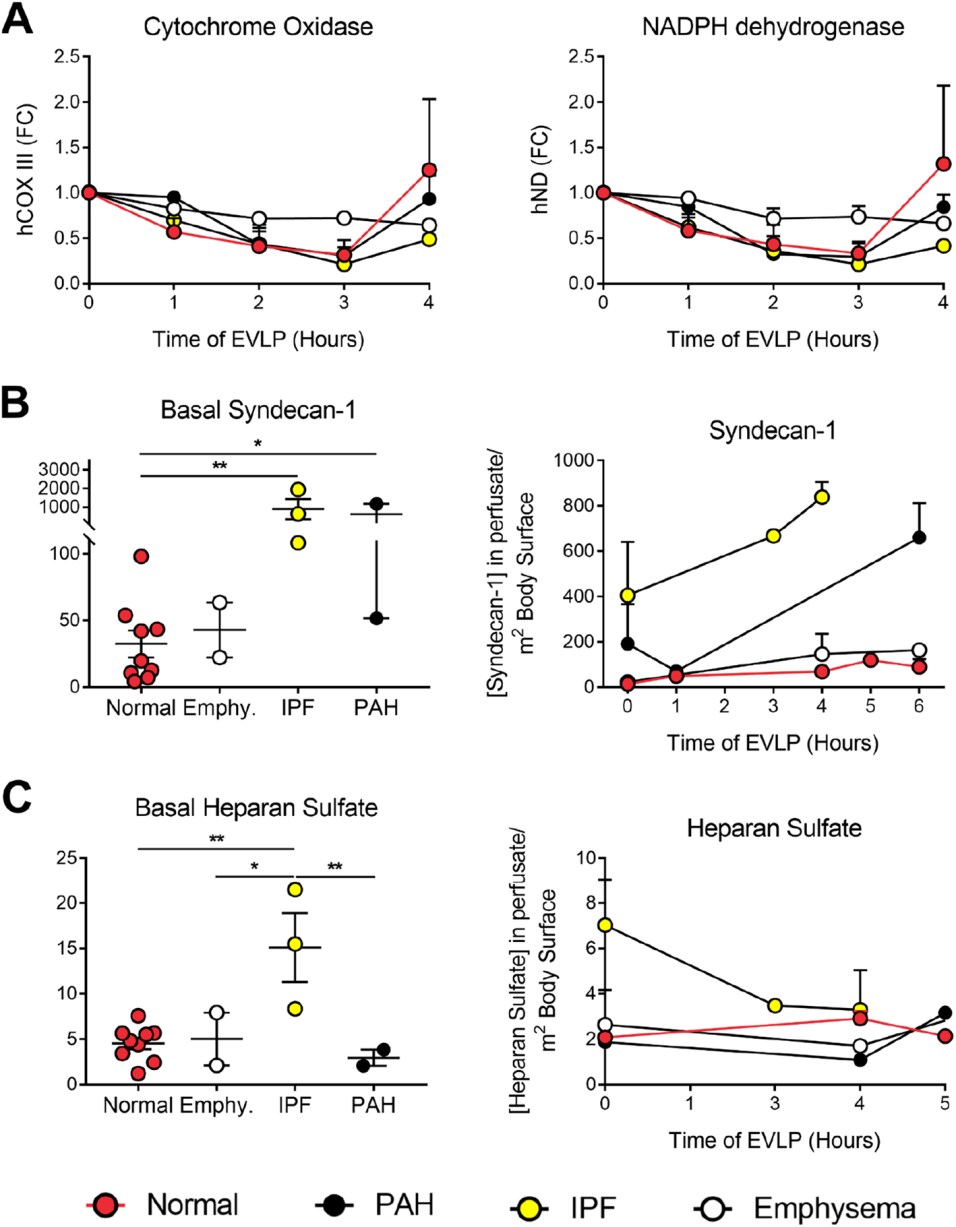
Soluble markers of cellular damage. (**A**) Mitochondrial DNA measured by qPCR of Cytochrome Oxidase and NADPH dehydrogenase, normalized to genomic DNA. The concentration of Syndecans (**B**) and Heparan sulfate (**C**) mRNA analysis of perfusate fluid measured by ELISA shown basal levels before EVLP (left) and through the course of the EVLP (right). *p < 0.05; **p < 0.01.

It is known that the release of mitochondrial DNA into circulation is a biomarker of cellular damage on different pathologies of acute and chronic lung diseases. We investigated if the ventilation and perfusion during EVLP led to lung injury within the donor's lungs at the transcriptomic level by measuring mitochondrial genes for COX III and hND. We measured by qPCR the levels of COX III and hND DNA transcripts in the perfusion solution. We observed a decrease in COX III and hNDin all the samples collected in all the groups during the three first perfusion hours. Interestingly, we observed a non-significant increase, only in control and in a lower proportion in the PAH samples, at four hours on the levels of DNA transcripts for the two genes in circulation (Fig. 2A).

Maintenance of endothelial integrity and the alveolar-capillary barrier has been the cornerstone of donor organ preservation for optimal functioning. The several events occurring during inflammation are modulated by chemokines, integrins, and selectins, and their interaction with glycosaminoglycans, mainly heparan sulfate^31^. We, therefore, measured syndecan-1 (Fig. 2B) and heparan sulfate (Fig. 2C) concentrations at baseline and in the perfusate during EVLP, as indicators of injury and inflammation, which is mostly related to glycocalyx shedding that is the first step in endothelial dysfunction; we observed that lungs form PAH and IPF, which have the most significant damage of the endothelial, have higher glycocalyx in the perfusion solution.

**Figure 3:**
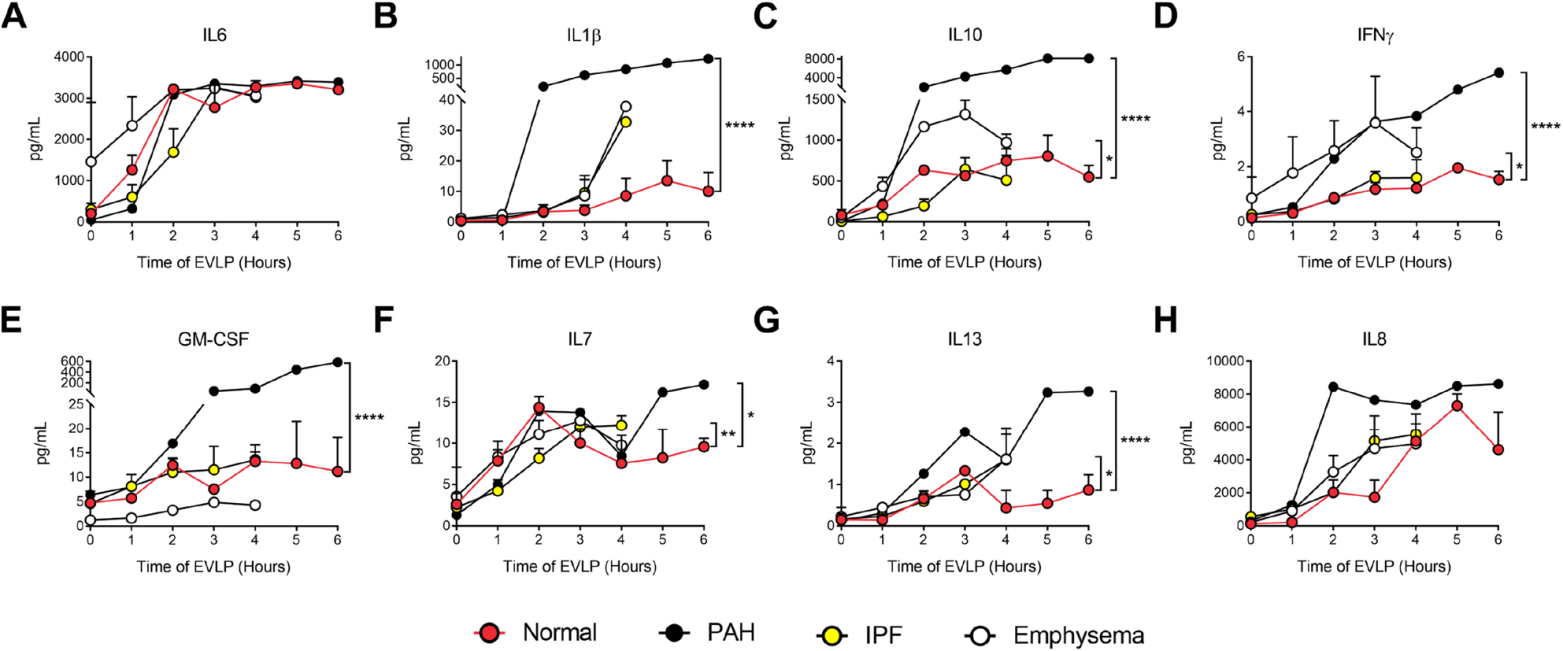
Cytokine measurements performed by multiplex ELISA are displayed for IL-6, IL-1β, IL-10, IFNγ, GM-CSF, IL-13, IL-7, and IL-8 (**A-H**). *p < 0.05; **p < 0.01; ****p < 0.0001.

To test inflammatory responses to EVLP, we checked a panel of inflammatory cytokine levels in the perfusate. The proinflammatory cytokine expression of Interleukin IL-1β, INFγ, GM-CSF, IL-13, IL-7, and IL-8 significantly increased in PAH samples with peak levels seen after 2 h of the onset of EVLP. Interestedly, anti-inflammatory IL-10 was one of the cytokines that had one of the highest increases. We did not observe differences between the groups for the kinetics of IL-6, suggesting the increase in IL-6 is dependent on the mechanics of the EVLP (Fig. 3A–H).

### Differential gene expression (DEG) for various chronic lung diseases on EVLP

The EVLP technique emerged as a means for organ preservation and repair pre-transplantation, in which the organ is kept biologically active at the tissue and cellular levels. It has been demonstrated that biological signals are triggered with the organ perfusion, which allows for organ optimization before its transplantation.

**Figure 4:**
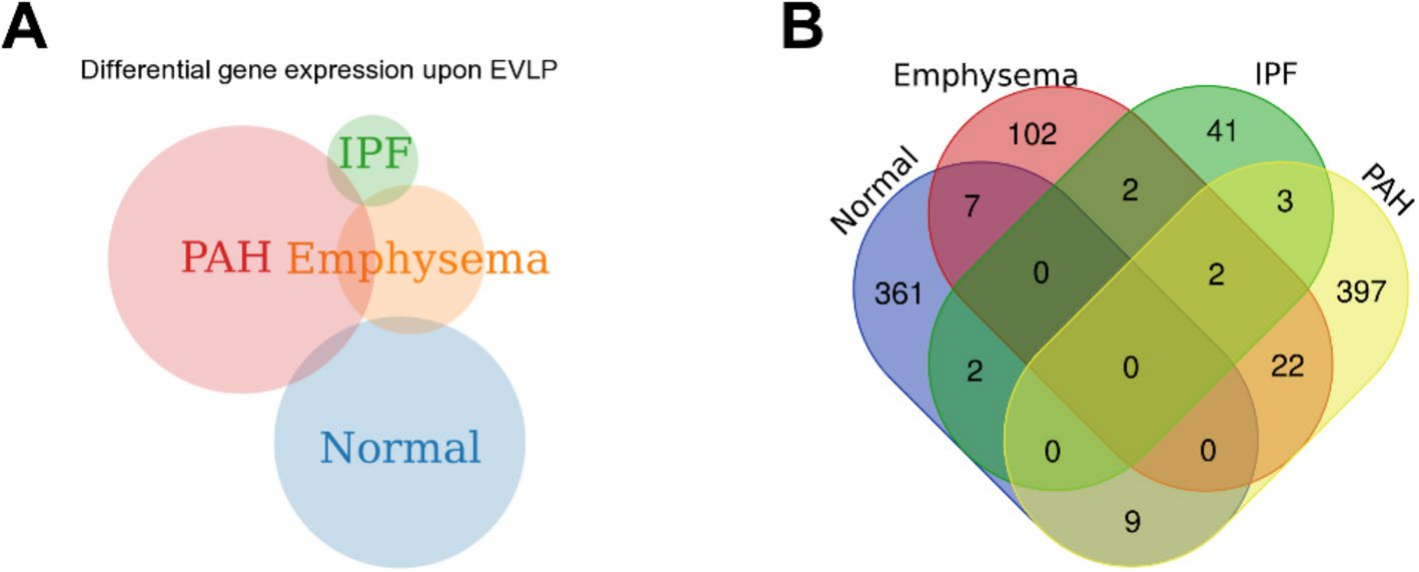
Differential Gene expression (DEG) in lung tissue biopsies taken from PAH, IPF, Emphysema, and Normal lungs at time 0 h vs. 6 h during EVLP expressed as Venn diagrams. (**A**) Shows a Venn diagram with proportional representation of the number of genes, and (**B**) shows the number of DEG in each of the intersections.

**Figure 5:**
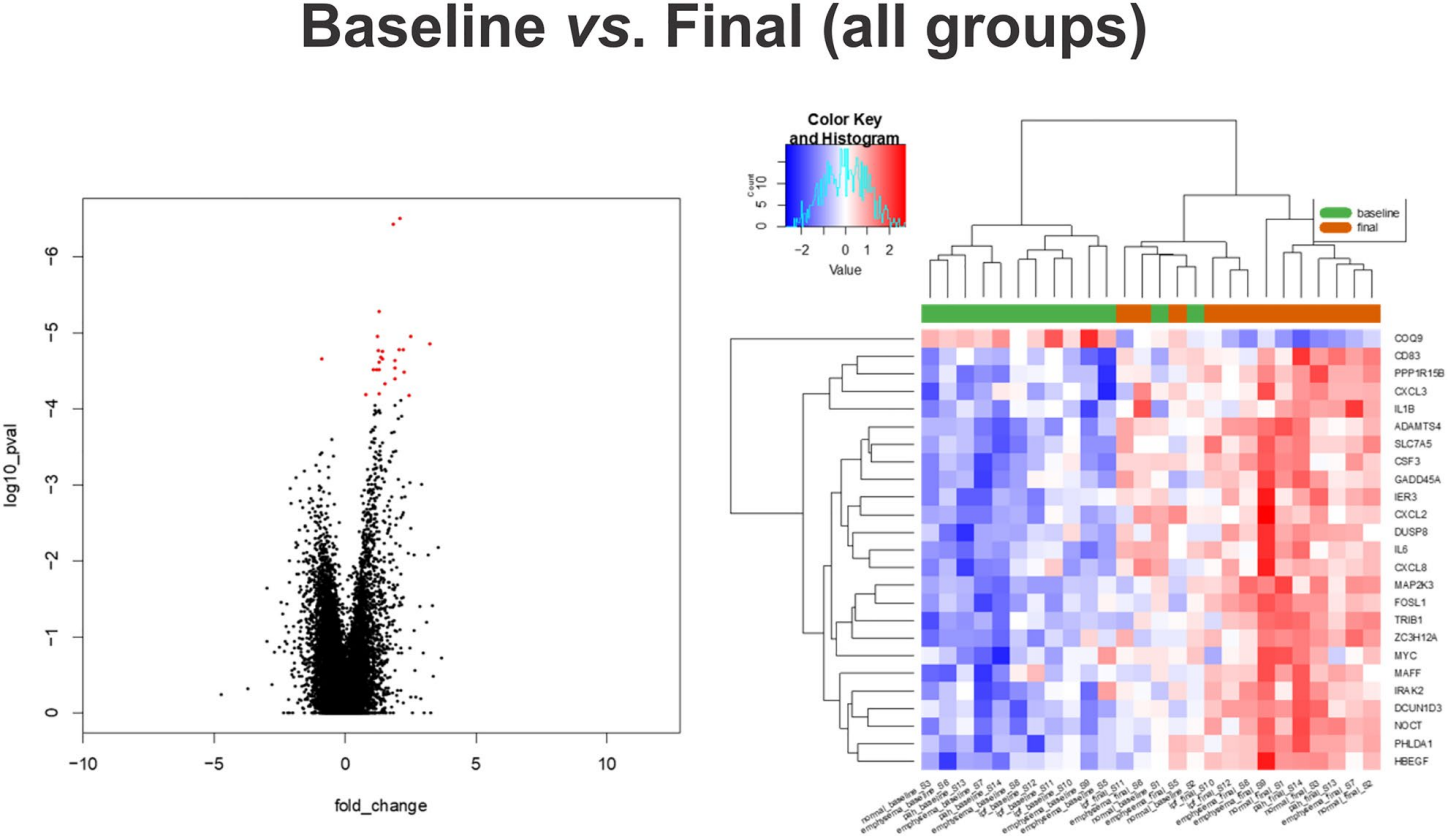
Heatmap and volcano plot of differentially expressed genes before and after EVLP of all lung samples. FDR adjusted p value < 0.05.

**Figure 6:**
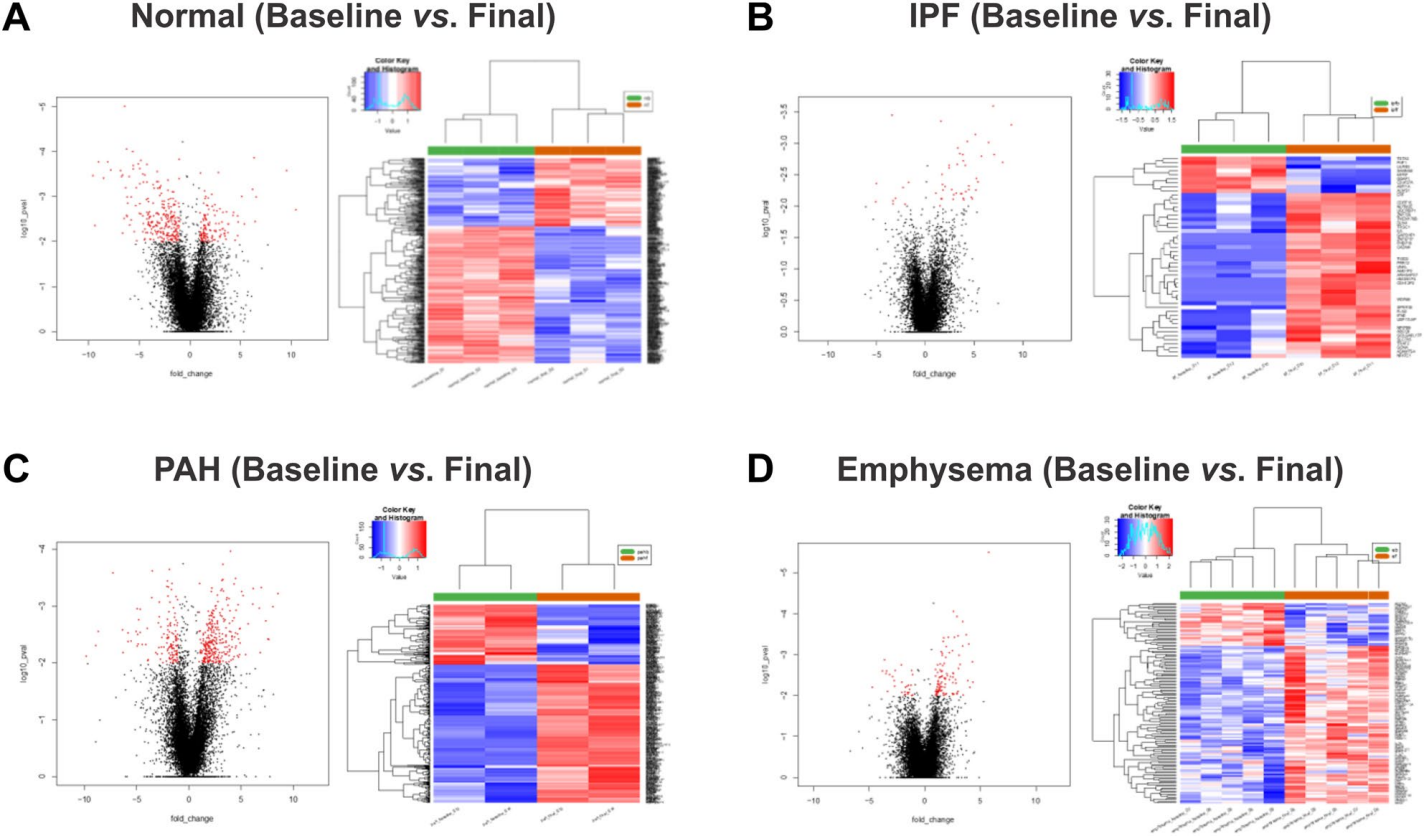
Heatmap and volcano plots of differentially expressed genes before (green) and after EVLP (orange) in normal (**A**), IPF (**B**), PAH (**C**), and Emphysema (**D**). Raw p value < 0.01 and |logFC|> 1.

We used RNA-seq of lung biopsies taken before and after EVLP to perform DEG and pathway analysis. We sought to explore which pathways were impaired or activated upon EVLP when the lungs are already in a chronic disease state by comparing the pre- and post- EVLP enriched pathways in each group. EVLP triggered the differential expression of 379 genes in healthy lungs, 433 in PAH, 135 in emphysema, and 50 in IPF (Figs. 4, 5, 6). This is consistent with the lungs' observations at the histological levels, in which PAH presents with most histopathological changes and highest amounts of proteinaceous depositions, and IPF lungs suffer the least histological changes with EVLP (Fig. 1B).

Two genes were commonly upregulated in all diseases with EVLP but not in healthy lungs, being mostly expressed in PAH and least in IPF: Interleukin-6 (IL-6; p < 0.01) and Solute Carrier Family 7 Member 5 (SLC7A5 or LAT1; p < 0.01). However, while IL-6 levels in circulating perfusate increased over time, we did not observe significant changes than normal lung EVLP (Fig. 3A).

**Figure 7:**
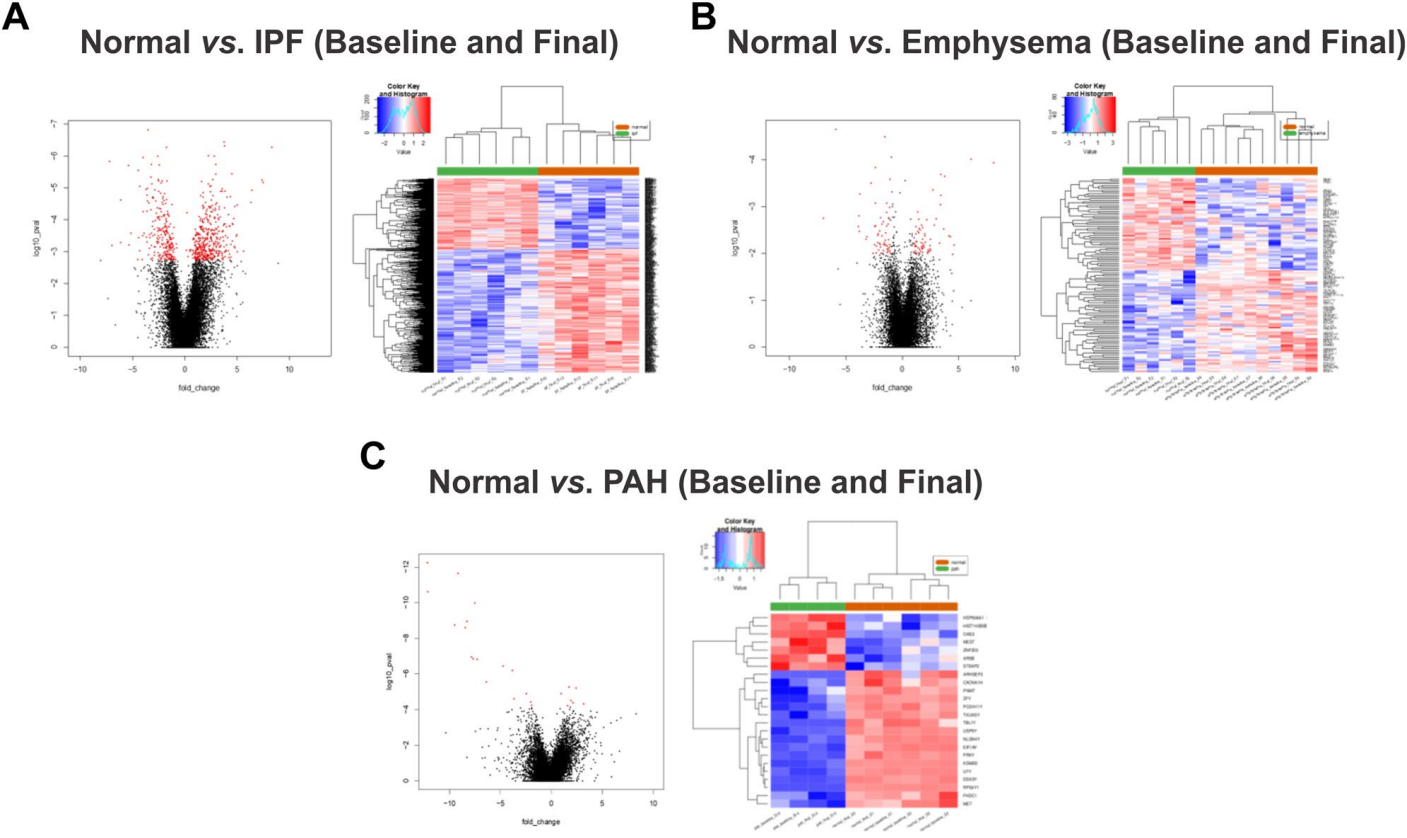
Heatmap and volcano plots of Differentially Expressed Genes before and after EVLP in IPF (**A**), Emphysema (**B**), and PAH (**C**) (green) compared to Normal (orange). Raw p value < 0.01 and |logFC|> 1.

Three uncharacterized genes were differentially expressed upon lung perfusion in PAH and IPF: Tigger Transposable Element-Derived protein 3 (TIGD3), a DNA-binding protein-coding gene (> 2.4 FC; p < 0.005), and ENSG00000262660 and ENSG00000262730, both of which were downregulated in PAH and upregulated in IPF. PAH and emphysema were the two groups that were modified most similarly upon EVLP in terms of gene expression, sharing 22 genes modified in the same direction. Among these, there is upregulation of genes involved in inflammatory processes, such as cytokines IL-6 and IL-1β (Table 3, Fig. 7), which concentrations increased in the circulating perfusate in all diseases showing significantly higher concentrations of IL-1β compared to normal (Fig. 3A,B).

**Table 3:** Fold change of genes changed with EVLP (T6 *vs*. T0).

Gene	PAH	Emphysema	IPF
	logFC		
*IL6*	4.888332402	4.334075211	2.086902953
*SLC7A5*	2.677956466	2.286980019	1.574568482
*ST13P11*	4.734050096	2.964897713	
*CXCL8*	4.575172644	4.382958296	
*CCL20*	4.185392968	3.211892607	
*IL1A*	3.599585349	3.451140607	
*EREG*	3.218835227	2.81601774	
*FOSL1*	3.218835227	2.681673531	
*DUSP8*	3.039281662	2.54183477	
*IL1B*	2.805667735	3.076751303	
*CD83*	2.390624885	1.362090484	
*TRIB1*	2.142510594	1.259076891	
*PHLDA1*	2.133756578	1.642541373	
*HBEGF*	2.072389405	1.818738561	
*CXCL2*	2.030794889	3.444701422	
*PPP1R15B*	1.99853045	1.107213136	
*PLK3*	1.88294951	1.337666853	
*IFRD1*	1.860928973	1.274942625	
*STX11*	1.746644478	1.401319102	
*TUFT1*	1.666247994	1.867420107	
*HSPB8*	1.549575127	1.564186159	
*SAMD8*	1.066574423	1.160985625	
*MST1L*	− 2.648103998	− 1.67069406	
*ENSG00000284320*	− 4.788656866	− 2.685109758	
*TIGD3*	2.433881665		2.500381008
*ENSG00000262660*	− 2.054711105		3.409785589
*ENSG00000262730*	− 6.24815688		7.150325871
*ADAMTS4*		2.64216731	1.884242887
*NFATC1*		1.040314545	1.157756576

GSEA analysis revealed the enrichment of pathways upon perfusion specific for only Emphysema and IPF lungs among the diseased lungs, and all other pathways were enriched in all groups. Emphysema lungs showed the enrichment of the ‘Androgen response’ signaling dataset, probably due to the predominance of male donors in the group (75%, Table 1). Remarkably, IPF showed an enrichment of the downregulated genes in KRAS signaling (KRAS Signaling down gene-set from the Hallmarks database), and a downregulation of the variant 1 Myc targets and downregulated genes in UV response.

When comparing diseased lungs to healthy lungs after EVLP, which describe the gene expression differences inherent of the disease at the end of the EVLP, but not necessarily changed with the perfusion, IPF showed downregulation of 32 datasets. In contrast, PAH lungs showed the positive enrichment of three datasets: 'Allograft rejection,' 'Interferon-γ response,' and ‘Interferon-α response.' emphysema lungs did not show any difference in enrichment than healthy lungs after EVLP (Table 4).

**Table 4:** Pathways identified to be upregulated or downregulated (bold letters) with EVLP.

Normal	Emphysema	IPF	PAH
TNFα Signaling via NFκB	TNFα Signaling via NFκB	TNFα Signaling via NFκB	TNFα Signaling via NFκB
Apoptosis	Apoptosis		Apoptosis
Hypoxia	Hypoxia		Hypoxia
IL2/STAT5 Signaling	IL2/STAT5 Signaling		IL2/STAT5 Signaling
TGFβ signaling	TGFβ signaling		TGFβ signaling
UV response up	UV response up		UV response up
Allograft rejection			Allograft rejection
IL6/JAK/STAT3 Signaling			IL6/JAK/STAT3 Signaling
Inflammatory response			Inflammatory response
Interferon-α and γ responses			Interferon-α and γ responses
KRAS signaling up			KRAS signaling up
MTORC1 signaling			MTORC1 signaling
Oxidative phosphorylation		Oxidative phosphorylation	
P53 pathway			P53 pathway
Unfolded protein response			Unfolded protein response
Coagulation and complement			
E2F targets			
Epithelial-mesenchymal transition			
G2M checkpoint			
Myc targets V2			
PI3K Akt MTOR signaling			
Reactive oxygen species pathway			
	Androgen response		
		KRAS signaling down	
		**Myc targets V1**	
		**UV response down**	

**Figure 8:**
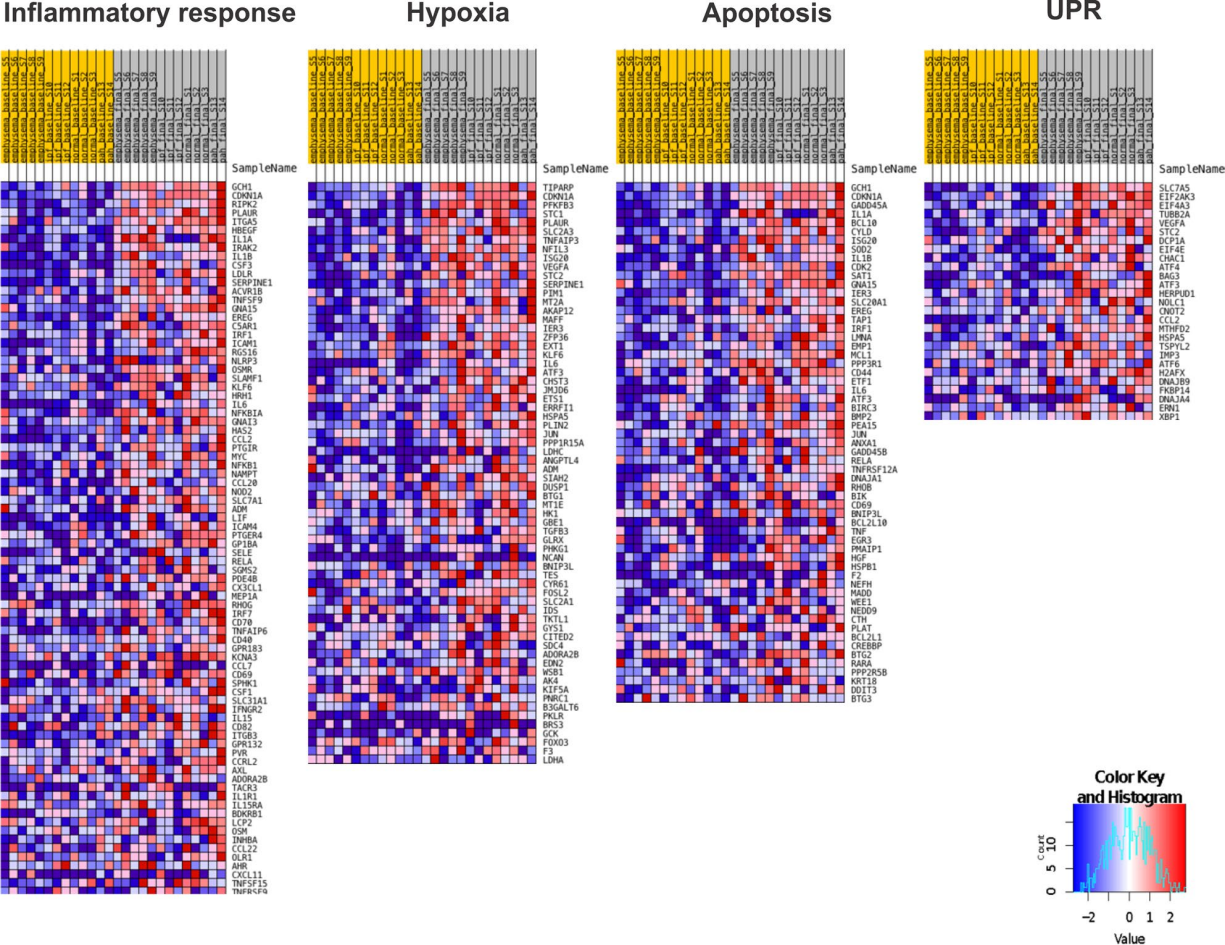
Heat maps for differentially expressed genes (DEG) in Inflammatory Response, Hypoxia, Apoptosis, and UPR pathways. Top differentially expressed genes in the Inflammatory Response, Hypoxia, Apoptosis, and UPR pathways at baseline (yellow) and final state (grey) of EVLP for all group samples (Normal, Emphysema, IPF, and PAH). (**A**, **C**) FDR adjusted p value < 0.05. (**B**) raw p value < 0.01 and |logFC|> 1.

To confirm the activated pathways during EVLP, we performed gene set enrichment analysis (GSEA) comparing all lungs pre- and post-EVLP. As described previously, we detected the immune system's activation upon EVLP with the enrichment of pathways such as 'TNFα signaling via NF-κB,' 'Inflammatory response,' 'IL-2-STAT5 signaling’ and ‘IL-6-JAK-STAT3 signaling'. Additionally, EVLP activated pathways involved in cell survival and response to stress, such as 'Apoptosis,' 'Hypoxia,' 'p53 pathway', 'Unfolded Protein Response' (UPR), or the upregulation of genes in response to UV radiation (Fig. 8).

## Discussion

The EVLP technique is broadly used as a procedure for the assessment and reconditioning of donor lungs initially considered unsuitable for transplantation. Improving physiological performance and reducing oxidative stress caused by the ischemia to which the lungs are subjected at the time of procurement the cornerstone of this recovery. Because of the limited number of donors of human lungs available for research EVLP, the large majority of the published work is mostly using organs from large animal models like pig and sheep. We and others have demonstrated that human EVLP is a unique platform for preclinical studies in an acute setting. This technique has been instrumental for the study of small-molecules^11^, gene-based therapy^32^, as well as a mesenchymal stem cell (MSC) and MSC-derived extracellular vesicles in acute lung injury^33^. However, this has been mostly tested in the context of very acute models, such as LPS-induced ARDS.

With this study, we demonstrate, for the first time, the feasibility of using EVLP to investigate several critical aspects of human pulmonary physiology in non-disease and multiple chronic lung diseases. In our preparations, we observed physiological correlations to know pathophysiology in the setting of chronic lung disease. PAH lungs had high PVR, and emphysema had high lung compliance IPF had low lung compliance, meanwhile PAH lungs had compliance in physiological ranges. The versatility of this preparation allows one to capture a wide range of data in a kinetic fashion. As an example is the case of the kinetics of Syndecan during perfusion. Our findings suggest changes in the endothelium that result in different levels of inflammation edema and histology injury. These findings indicate that Syndecan can be considered a possible biomarker of endothelium damage during ex vivo perfusion.

This work does not intend to recapitulate the biochemical responses triggered during disease development after years of progression. Contrary, our goal was to develop laboratory models that aid in evaluating therapeutic interventions in diseased lungs. Our data provide the first step of changes needed to current ex vivo lung perfusion preparations to evaluate diseased human lungs. Also, our goal was to capture physiological, biochemical, and genetic changes under baseline EVLP conditions of human disease lungs.

Each disease presents with the lung's unique structural remodeling, such as loss of the alveolar structure in emphysema, the heterogenic accumulation of fibrotic tissue in IPF, or the characteristic vasoconstriction and vasculature remodeling of PAH, all of which require different perfusion and ventilator approaches. Furthermore, because the lungs are at the end-stage of the disease, the time they can be subjected to EVLP might be limited. Here we show that we can successfully assess these lungs for a period of4 to 6 h of lung perfusion. Gas exchange, including oxygenation, was preserved, the lungs remained mechanically functional with reasonably good compliance, there was no evidence of increased cell death, and a protective genetic response was induced^34^. Also, we recognize that in the case of IPF lungs, in which there is a more severe compromise of the whole lung parenchyma, the capability to maintain the lung under ex *vivo* perfusion conditions is highly compromised. In several of the experiments in which IPF lungs were perfused and ventilated, the lung's integrity was maintained only up to 4 h. As our work continues, future changes to our perfusion/ventilation strategy may allow for longer perfusion times.

The inflammatory response to EVLP was significantly higher in the PAH lungs than the cytokine profile in the perfusate, confirmed by the histological evaluation. IL-6 levels in the perfusate were equally increased in all groups, albeit at the gene expression level, we report a significant increase of the IL-6 gene in chronically diseased lungs after ex vivo lung perfusion, compared to healthy lungs. The observed changes in the inflammatory cytokines GM-CSF, IFN-γ, IL-1β, IL-7, and IL-13 likely represent a stress response in PAH lungs. These data suggest that parenchyma cells are the primary source of proinflammatory cytokines because of the absence of circulatory inflammatory cells in the perfusate. It is worth noting that in high flow EVLP, the lungs' metabolic activity drives lactic acid production and downstream changes leading biochemical changes in the perfusate, which could potentially harm organ function over a longer circuit run. Therefore, rigorous monitoring and buffering remain the cornerstone during EVLP preparations.

Our findings demonstrate the expected high pressures in vascular resistance with PAH lungs' perfusion and not in the other groups. Interestingly, the results we present here give further insight into the pathophysiology and transcriptomic changes in vivo. Although the pulmonary pressures dictated the flow rates in these lungs, this maneuver resulted in decreased pulmonary artery pressure and peak airway pressures, which may have enhanced clearance of edema fluid with lower flow and a successive drop in the airway or alveolar resistances. There are, however, several limitations to use this technique for physiological measurements. The PVR values we report here (~ 880 dynes/sec/cm^5^ units in non-PAH) are elevated compared to typical human values (< 160 dynes/s/cm^5^). We think this could be due to systemic factors, as the EVLP pump and tubing add resistance to the circuit. The non-pulsatile nature of flow in the system and the lack of preload on the LV could have confounded our ability to derive physiologic mean, systolic, and diastolic pressures.

The decreased perfusion flow in PAH almost certainly led to the observed metabolic changes in measured glucose consumption with changes in bicarbonate levels compared to other preparations. In the gene array, a baseline genetic signature for each chronic disease was established, and during the stress of EVLP, there was a shared and differential gene induction compared to that from healthy lungs.

Due to animal models' inability to fully replicate disease states' human physiology, presenting with inherent limitations at predicting individual results from the experimental models, the EVLP technique has emerged as a promising platform to test human diseases in a close-to human physiological setting.

The use of non-disease lungs in EVLP has proven to be a useful and unique platform for acute preclinical studies in LPS-induced ARDS to evaluate small-molecules^11^, gene-based therapy^32^, as well as mesenchymal stem cells and their derived extracellular vesicles^33^.

To our knowledge, this is the first study to report the feasibility of EVLP for assessing various chronic lung diseases, such as PAH, IPF, and emphysema, especially with the use of genomic analysis. We believe this data will help EVLP to continue to establish itself as a translational medicine platform in end-stage lung disease, direct therapies, and provide a potential translational research opportunity.
